# Analysis of Multiple B-Value Diffusion-Weighted Imaging in Pediatric Acute Encephalopathy

**DOI:** 10.1371/journal.pone.0063869

**Published:** 2013-06-03

**Authors:** Yasuhiko Tachibana, Noriko Aida, Tetsu Niwa, Kumiko Nozawa, Kouki Kusagiri, Kana Mori, Kazuo Endo, Takayuki Obata, Tomio Inoue

**Affiliations:** 1 Research Center for Charged Particle Therapy, National Institute of Radiological Sciences, Inage-ku, Chiba, Japan; 2 Department of Radiology, Yokohama City University Graduate School of Medicine, Kanazawa-ku, Yokohama, Japan; 3 Department of Radiology, Kanagawa Children’s Medical Center, Minami-ku, Yokohama, Japan; 4 Department of Radiology, Tokai University School of Medicine, Isehara, Japan; Beijing Normal University, China

## Abstract

Acute encephalopathy is a disease group more commonly seen in children. It is often severe and has neurological sequelae. Imaging is important for early diagnosis and prompt treatment to ameliorate an unfavorable outcome, but insufficient sensitivity/specificity is a problem. To overcome this, a new value (fraction of high b-pair (F_H_)) that could be processed from clinically acceptable MR diffusion-weighted imaging (DWI) with three different b-values was designed on the basis of a two-compartment model of water diffusion signal attenuation. The purpose of this study is to compare F_H_ with the apparent diffusion coefficient (ADC) regarding the detectability of pediatric acute encephalopathy. We retrospectively compared the clinical DWI of 15 children (1–10 years old, mean 2.34, 8 boys, 7 girls) of acute encephalopathy with another 16 children (1–11 years old, mean 4.89, 9 boys, 7 girls) as control. A comparison was first made visually by mapping F_H_ on the brain images, and then a second comparison was made on the basis of 10 regions of interest (ROIs) set on cortical and subcortical areas of each child. F_H_ map visually revealed diffusely elevated F_H_ in cortical and subcortical areas of the patients with acute encephalopathy; the changes seemed more diffuse in F_H_ compared to DWI. The comparison based on ROI revealed elevated mean F_H_ in the cortical and subcortical areas of the acute encephalopathy patients compared to control with significant difference (P<0.05). Similar findings were observed even in regions where the findings of DWI were slight. The reduction of mean ADC was significant in regions with severe findings in DWI, but it was not constant in the areas with slighter DWI findings. The detectability of slight changes of cortical and subcortical lesions in acute encephalopathy may be superior in F_H_ compared to ADC.

## Introduction

Acute encephalopathy is a generic term for brain dysfunction of acute onset that often occurs subsequent to infectious diseases with fever, such as influenza and human herpes virus type 6 [1], [2]. It is most common in infants and young children, is manifested clinically with stupor/coma and febrile seizure, and is often severe and prolonged [3]. Magnetic resonance imaging (MRI), especially high signal intensity in diffusion-weighted images (DWI), is known to be useful for detecting brain lesions [4]–[10]. Recently, several subtypes of acute encephalopathy have been categorized on the basis of MRI findings and clinical manifestations: acute necrotizing encephalopathy (ANE) [11], [12], hemorrhagic shock and encephalopathy syndrome (HSES) [2], clinically mild encephalitis/encephalopathy with reversible splenial lesion (MERS) [10], and acute encephalopathy with biphasic seizures and late reduced diffusion (AESD) [6], [8], [10]. The characteristic findings, outcome, recommended treatment and genetic background for each subtype are gradually becoming clear [2], [7], [9], [10], [12]–[17], but the pathological mechanisms are still uncertain, and many cases of acute encephalopathy are unspecific and could not be directly categorized into the above subtypes [1], [3], [7], [15].

The outcome of acute encephalopathy, except MERS, is often unfavorable [1], [2], [7], [9], [12]. Neurologic sequelae and even death are quite common. Diagnosis and treatment (e.g. steroid, human immunoglobulin and hyperthermia [2], [10]) in the early stage are assumed to be crucial for ameliorating brain damage [5], [9]. In this respect, rapid diagnosis by MRI is very important, but the findings, as aforementioned, are often unspecific, and they change remarkably with the time course even in typical cases. For example, reduced subcortical water diffusion is mentioned as an important hallmark of AESD, but it is also found in various brain lesions including other categories of (or uncategorizable) encephalopathies [5]. In addition, this finding in AESD is found best at 3–9 days from onset, but usually not in the earlier days or the later days (mild reduced diffusion in cortex may be found instead) [6], [8], [10]. For these reasons, both sensitivity and specificity of the DWI findings are not sufficient at present.

The reduced diffusion of acute encephalopathy was previously discussed on the basis of visual assessments of DWI, which is intrinsically related to the apparent diffusion coefficient (ADC) calculated by the following monoexponential equation using two different b-values:
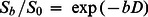
(1)where S_b_ and S_0_ indicate the signals with and without diffusion sensitizing gradients, b indicates the b-value, and D indicates ADC. However, the calculated values are sometimes misleading when applying different b-values in DWI because the signal attenuation does not always follow Eq. 1 in vivo. To compensate for this limitation, another model of signal attenuation that considers two separate diffusion components (fast and slow components) with exchange has been well discussed [18]–[27]. This two-compartment model is given by this biexponential equation:
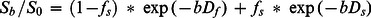
(2)where fs indicates the fraction of the slow diffusion component, and Df and Ds indicate ADC of each fast and slow diffusion component, respectively. This equation (Eq. 2) is known to fit the experimental signal attenuation better than Eq. 1, and thus is often adopted for discussing in vivo water diffusion.

The detailed construction of the two compartments and their kinetics (balance, exchange) are not well established, but the parameters D_f_, D_s_, and f_s_ have been discussed previously to characterize tissue properties in a pathologic context of the human brain, such as ischemia, edema, tumor, and post-radiation [18], [20], [21], [28]. Application of this model to the prostate [23] and mammalian glands [24], [25] was also reported. However, it is not easy to calculate the exact fraction (f_s_) in clinics because it requires a relatively large amount of data acquired from many different b-values ( = longer scan time), a circumstance particularly unsuitable for acute encephalopathy patients, as it is risky, because of the disease severity, to stay in the MRI apparatus for an extended period. In addition, longer scan time directly increases the risk of failing the scan, as the duration of needing to remain still is also necessarily prolonged. This general problem in children can usually be managed by sedation agents, but seizures that can occur in acute encephalopathy might require extra doses, which also pose a risk for the severe state.

In this study, we designed a more simplified value “fraction of high b-pair (F_H_)” related to the fraction of the two compartments. The purpose of this study is to compare F_H_ with ADC in terms of the detectability of pediatric acute encephalopathy, and to discuss its significance.

## Materials and Methods

### Ethics Statement

This retrospective study was approved by the ethics committee of the Kanagawa Children’s Medical Center, where all of the clinical data in this study was acquired. Written informed consent was waived by the ethics committee.

### Subjects

We searched our brain MRI database for pediatric patients (under 12 years old) who had undergone the four b-value diffusion-weighted imaging sequence (4bDWI, described later) as part of their clinical examination, and then designed two clinical groups for comparison: control group and encephalopathy group. The control group included patients who had undergone MRI examination for their neurologic symptoms but no abnormal imaging findings including 4bDWI had been found, and also whose symptoms were transient and disappeared without sequelae. The encephalopathy group included patients who had ultimately been diagnosed with acute encephalopathy based on their clinical findings in addition to the abnormal DWI high intensity area found in their cerebrum. The clinical findings included continued consciousness insufficiency for at least 24 hours except sedation for hypothermia and/or intubation. Only patients with unfavorable outcome (death, or remaining neurologic sequelae such as impaired consciousness, spasticity) were included in the encephalopathy group (thus, patients with MERS were not included), and if MRI had been performed within 7 days from onset. For patients diagnosed as AESD, a secondary seizure was considered as the onset for this criterion.

For both groups, patients less than one year old were excluded to avoid the heterogeneity of myelin development, which it may affect water diffusion. Patients with metabolic diseases, prominent cerebral dimorphisms (e.g. corticogyral malformation, diffuse volume loss of brain from some other previous disease, chronic infarction), and severe motion affection on 4bDWI were excluded from both groups. Finally, 16 (1–11 years old, mean 4.89, 9 boys, 7 girls) and 15 patients (1–10 years old, mean 2.34, 8 boys, 7 girls) were selected for the control and encephalopathy groups, respectively. Indications for MRI examination of the control group were convulsion with a fever over 38°C (n = 4), convulsion (n = 2), suspicion of slight cognitive or motor impairment (n = 5), and other unspecific symptoms (n = 5). Definite diagnoses of the encephalopathy group were AESD (n = 5), ANE (n = 1), and other nonspecific encephalopathies (n = 9).

### MR Imaging

All MR imaging was performed using a 3T MRI system (Verio 3T; SIEMENS, Germany) with either a 12-channel head coil or a combination of two 4-channel flex coils, depending on head size and age. Patients younger than 6 years and/or uncooperative regarding staying still were sedated during the imaging. Routine clinical MR imaging sequences in our institution consisted of transverse and sagittal T1-weighted spin-echo imaging, and transverse and coronal T2-weighted fast spin-echo imaging. Additional planes and sequences, such as fluid-attenuated inversion recovery imaging, diffusion-weighted imaging (b-value at 1000 sec/mm^2^), susceptibility-weighted imaging, contrast enhanced T1-weighted spin-echo imaging, and magnetic resonance spectroscopy were obtained depending on the disease and the patient’s condition. 4bDWI was also performed as an additional sequence when the patient was believed to have abnormalities that might not have been assessed sufficiently by the normal routine DWI sequence, but only if the clinical situation was stable and the benefit of the examination was believed to exceed the risk of the longer scanning time. This point was decided by an experienced board-certified pediatric neuroradiologist (N.A., 27-year experience) assisted by a board-certified pediatric neurologist attending the MRI system during the entire examination.

#### Four b-value diffusion-weighted imaging sequence (4bDWI)

The outline parameters of the DWI sequence assessed in this study are shown in Table 1. All images were obtained in axial slices using the spin-echo-type single-shot echo planner imaging (EPI) technique. A parallel imaging technique for a multi-channel detector (GRAPPA: Generalized Autocalibrating Partially Parallel Acquisition) was applied. Motion probing gradients (MPG) were applied by Twin-Refocus-Spin-Echo technique. They were set in 3 orthogonal directions (anterior-posterior, left-right, cranio-caudal) separately, making three different image series. Trace DWI images, were then generated as their geographic mean. Imaging was automatically repeated four times to acquire four DWI image series with different b-values (the b-value was converted in every repetition, i.e., b = 0, 500, 1500, 2500 sec/mm^2^). Hence, four trace DWI image series with different b-values, respectively, were finally obtained.

**Table 1:** Outline of 4bDWI sequence.

MRI	3T MAGNETOM Verio, SIEMENS
Coil	12 ch head coil/4 ch flex coil
b-value	0, 500, 1500, 2500
TR/TE (msec)	7500/125
NEX	2
Matrix	Base 128 (Phase 70%)
FOV (mm)	200
Slice thickness (mm)	3
Band width (Hz/pixel)	1184
Scan time	2 min 54 sec

4bDWI: Four b-value diffusion-weighted imaging sequence, TR: repetition time, TE: echo time, NEX: number of excitations, FOV: field of view.

### Confirmation of Signal to Noise Ratio (SNR)

To confirm that there were enough SNR even in DWI images acquired at b = 2500 as described above, the following assessments were done in each patient of both groups. First, ROI was manually set at the center of the head of right or caudate nucleus; the b = 0 image was used. This procedure was performed by a board-certified radiologist. Then the ROI was automatically copied and also applied to the b = 2500 image. Finally, the mean signal intensity of the pixels included in the ROI at b = 2500 was divided by the standard deviation of their signal intensity at b = 0. The SNR was considered sufficient when this ratio was >3.0. The difference between the two groups was also assessed statistically using the Mann-Whitney test. A P-value <0.05 was considered significant.

### Calculation of F_H_

**Figure 1:**
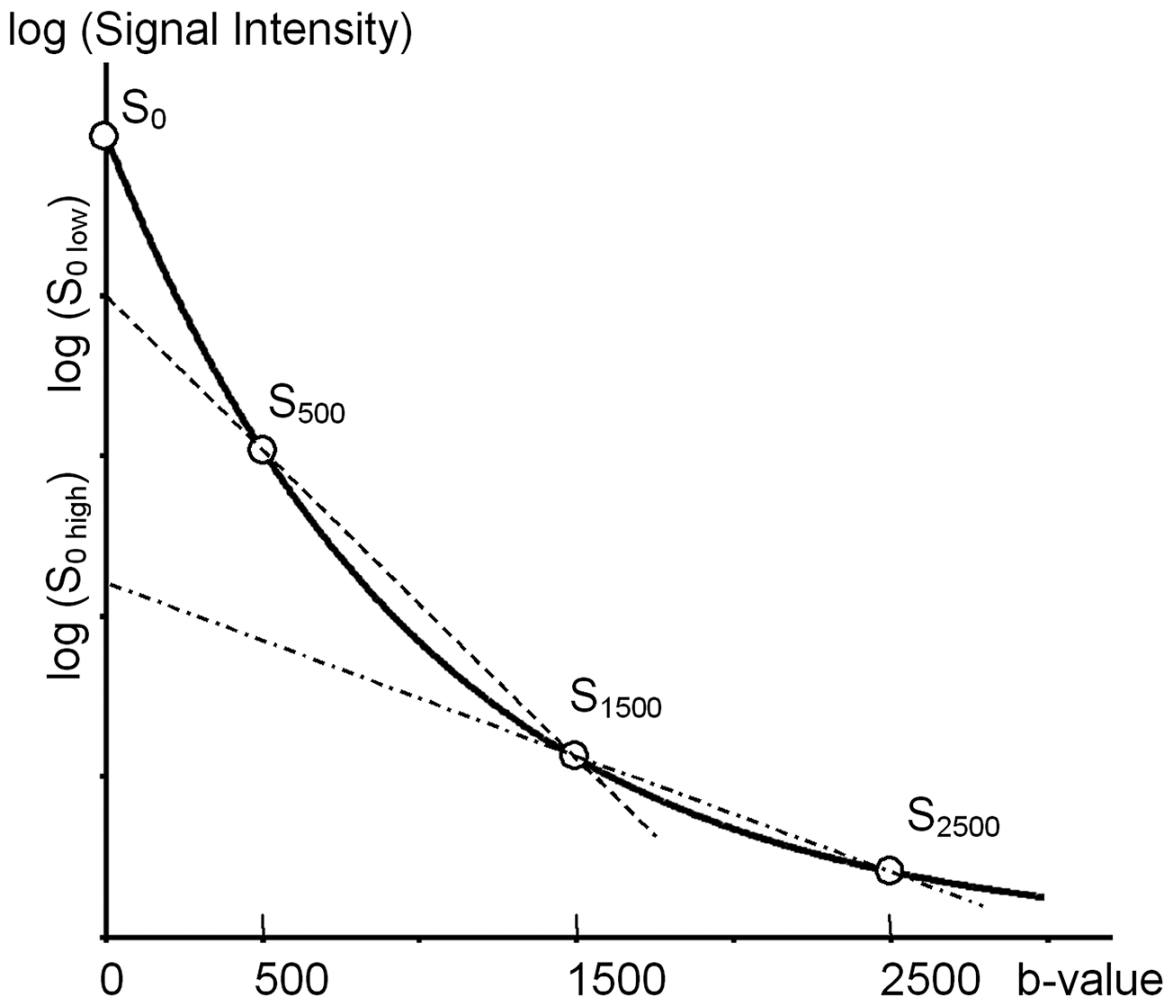
Calculation of F_H_. The graph illustrates typical in vivo signal attenuation of diffusion-weighted imaging (DWI). S_0_ to S_2500_ are the signal intensities of the corresponding b-values. S_0high_ and S_0low_ are the theoretical S_0_ values calculated from a different data pair (pair of S_500_ and S_1500_, and pair of S_1500_ and S_2500_, respectively) using a monoexponential fitting (which becomes a straight line in this semi-logarithmic graph). Fraction of high b-pair (F_H_) equals S_0high_/S_0low_.

F_H_ was calculated from the signal intensities of b = 500, 1500, 2500 (S_500_, S_1500_, S_2500_). First, two different theoretical signal intensities of b = 0 (S_0low_ and S_0high_) were calculated from the signal intensity of the lower b-value pair (S_500_, S_1500_) and the higher b-value pair (S_1500_, S_2500_), respectively, based on the assumption of monoexponential signal attenuation (Introduction, Eq. 1). The former equals the y-intercept of the line connecting S_500_ and S_1500_ on a semi-logarithmic graph (b-value as x-axis, and logarithm of signal intensity as y-axis), while the latter equals the y-intercept of the line connecting S_1500_ and S_2500_ on the same graph. Then, F_H_ was calculated by S_0high_/S_0low_ (Fig. 1).

### F_H_ Mapping

F_H_ was calculated pixel-by-pixel for each of the obtained 4bDWI data. Then, F_H_ was mapped on the original image data so that it could be evaluated just like the usual MR images in clinics.

We applied several masking steps to each slice of the F_H_ map to exclude background (air) pixels and cerebrospinal fluid (CSF) pixels. First, to exclude background pixels, we excluded pixels whose signal intensities of b-values = 0, 500, 1500, 2500 did not decline in this order. Pixels from noise might be excluded by approximately 96% (1– 1/(4!)) by this procedure, because their signal intensity should be in random order. Second, to exclude CSF pixels, we excluded the pixels from which the ADC calculated from the signal intensities of b = 0 and 1500 (monoexponential fitting (Introduction, Eq. 1)) was over 1.5×10^−3^ mm^2^/sec. Finally, binary neighborhood operations were applied to exclude the remaining background/noise pixels around the pixels of brain parenchyma; “erosion” and “dilation”, both based on the 4-connected neighborhood, were performed in this order. After these operations, smoothing of the remaining image (consisting mostly of the pixels of brain parenchyma) was done by mean filtering: each pixel value was replaced by the mean value of its 8-connected neighborhoods and itself (9 pixels in total). The pixels masked by the previous masking procedure were excluded if they were included in these 9 pixels. These mapping operations as well as the other operations in this study on the acquired DWI images were all performed using our in-house software developed with a commercial analysis package (Matlab version R2007b, MathWorks Inc., Natick, MA, USA).

### ROI-based Study

#### ROI setting on DWI images

**Figure 2:**
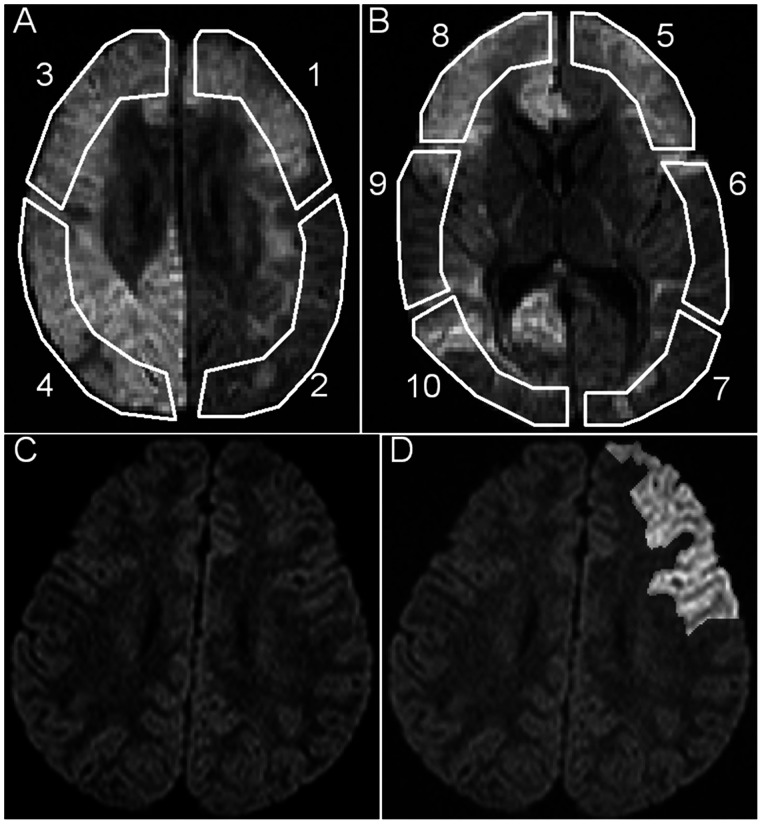
Regions of interest (ROIs) for the ROI-based study. A, B: Images acquired from a 1-year-old boy of unspecific acute encephalopathy. The images show the two specific slice levels selected for evaluation: the slice at the level of cerebral hemisphere (A), and the slice at the level of cerebral basal ganglia (B). Ten regions of interests (ROIs) were set as schematically illustrated for further assessments. C, D: A sample of accurate ROI 1 (frontal lobe of left cerebral hemisphere) acquired from a 2-year-old boy with suspected labium dyskinesia but which regressed a while after the examination (control group). C: Diffusion-weighted image before setting ROIs. D: Brightened area indicates the selected ROI, from which the pixels of cerebrospinal fluid and deep white matter were excluded as much as possible.

In each patient, two specific slices were first selected for evaluation: one slice at the level of the cerebral hemisphere, the most cranial slice in which both lateral ventricles are demonstrated, and the other at the level of cerebral basal ganglia, in which both the genu and the splenium of the corpus callosum are most broadly demonstrated. Second, the resolution of each image was increased to four times higher. Third, 10 ROIs in total were set on the slices (Fig. 2, A,B). Detailed criteria were as follows. All ROIs included only the cerebral cortex and subcortical white matter as completely as possible; ROIs 1 to 4 were set on the slice of the cerebral hemisphere; ROI 1: frontal lobe of left cerebral hemisphere, excluding internal cerebral cortex/subcortical white matter; ROI 2: temporal lobe of left cerebral hemisphere, excluding internal cerebral cortex/subcortical white matter; ROIs 3, 4: conformed to criteria of ROIs 1, 2 at right cerebral hemisphere; ROIs 5 to 10 were set on the slice of cerebral basal ganglia; first, the left cerebral hemisphere, excluding internal cerebral cortex/subcortical white matter, was selected; then the area was equally radially separated into three areas, according to the length along the brain surface; these regions corresponded to ROIs 5, 6, 7 in this order from rostral to dorsal; ROIs 8, 9, and 10: conformed to the criteria of ROIs 6, 7, 8 at the right cerebral hemisphere. To have the ROIs include only cortex and subcortical white matter as completely as possible, they were first manually set on DWI images (b = 1500) while being careful to include deep white matter as little as possible. Then, to exclude CSF pixels, the pixels from which the ADC (calculated from DWI images of b = 0 and 1500) was higher than 1.50×10^−3^ mm/sec^2^, were excluded automatically from the manually designed ROIs (Fig. 2, C,D). These ROIs designed on DWI images of b = 1500 were copied and also applied to DWI images of b = 0, 500, and 2500 automatically.

The averaged signal intensity of each ROI was also processed for each b-value separately (S_0_, S_500_, S_1500_, S_2500_ for each ROI, with the subscripted numbers corresponding to the b-values).

#### Visual assessment

**Figure 3:**
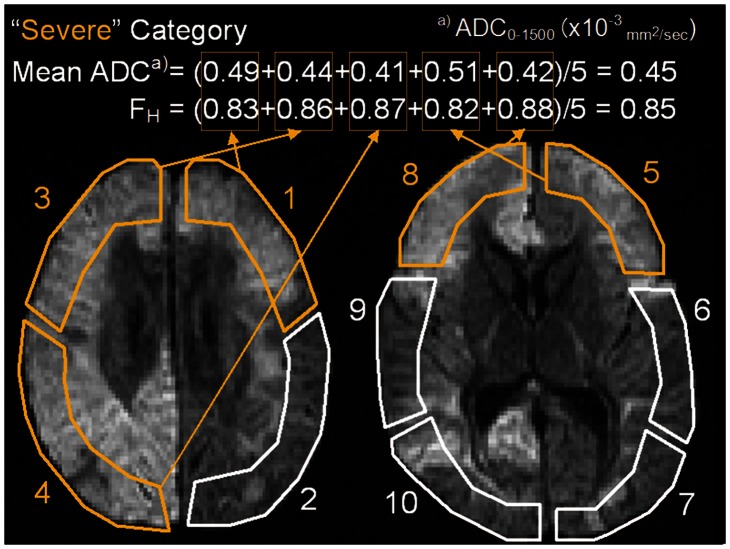
Mean F_H_ and ADC calculated in the encephalopathy group for analysis. Mean fraction of high b-pair (F_H_) and apparent diffusion coefficient (ADC) for statistics were calculated for each patient. For the patients in the encephalopathy group, mean F_H_ and ADCs were calculated for each category separately by averaging F_H_ and ADCs across the regions of interests (ROIs) corresponding to each category. In this case (same case as in Fig. 2), five ROIs (ROIs 1, 3, 4, 5, and 8) corresponded to the “Severe” category, so the mean F_H_ and ADC (only ADC calculated by b-value = 0 and 1500 (ADC_0–1500_) is shown here as example) for the “Severe” category was the averaged F_H_ and ADC of the five ROIs. In this case, ROIs 7, 9, and 10 were categorized as “mild” and ROIs 2 and 6 as “indistinct”. Note: the schematic ROIs illustrated on the images seem to include deep white matter and cerebrospinal fluid, but the accurate ROIs for assessments did not include these areas (Fig. 2; D).

In the encephalopathy group, the DWI images at b = 1500 were assessed visually. Each ROI was assessed separately in terms of prominence (strength) and expanse of abnormal signal intensity, and was categorized into three groups: severe, prominent abnormal signal intensity in more than 1/5 of the ROI; mild, prominent abnormal signal intensity between 1/10 and 1/5 of the ROI, or a slight abnormal signal intensity in more than 1/3 of the ROI; indistinct, prominence and expanse of abnormal signal intensity less than the above criteria (sample shown in Fig. 3). This categorization was first made by two board certified radiologists (Y.T. and T.O., 7-year and 20-year experience in MRI interpretation, respectively) independently. Both were blinded to any clinical information at the time of assessment. Finally, for ROIs for which categorizations differed between the interpreters, a final decision was reached by consensus.

#### Calculation of F_H_ and ADC

First, F_H_ was calculated for each ROI from the signal intensity of b = 500, 1500, and 2500 (S_500_, S_1500_, S_2500_). The calculation method was the same as previously described in the “Calculation of F_H_” section.

Second, ADCs were calculated for each ROI in four ways from the signal intensities of four different b-value pairs (S_0_ and S_1500_, S_0_ and S_500_, S_500_ and S_1500_, and S_1500_ and S_2500_), based on the assumption of monoexponential signal attenuation (ADC_0–1500_, ADC_0–500_, ADC_500–1500_, and ADC_1500–2500_; pairs of subscripted numbers are pairs of b-values used to calculate ADC). ADC_0–1500_ was included to represent the most commonly used ADC in clinics. Third, to arrive at the final values for statistics, mean F_H_ and ADCs for each patient were calculated. In the control group, mean F_H_ and ADCs were calculated by averaging F_H_ and ADCs across all 10 ROIs. In the encephalopathy group, mean F_H_ and ADCs were calculated for each category separately by averaging F_H_ and ADCs across the ROIs corresponding to the category (Fig. 3).

#### Statistical analysis

In the control group, the relations between patient age and F_H_ and between patient age and ADC_0–1500_ were assessed using Spearman’s rank correlation coefficient. Then, the mean F_H_ and ADCs calculated in the previous section for statistical purposes were compared between the control group and each category (severe, mild, and indistinct) of the encephalopathy group, respectively. Analysis of covariance (ANCOVA) was applied for these comparisons, with patient age being used as an additional variable. The P-values calculated by this procedure were tripled according to Bonferroni correction to avoid type 1 errors in the multiplicity of statistical analysis. A P-value (corrected in latter comparison) <0.05 was considered significant.

## Results

### Results of SNR Confirmation

The range and median of the ratio (i.e. mean signal intensity of b = 2500 divided by standard deviation of the signal intensity of b = 0) in the control group were: minimum, 4.36, maximum, 9.89, median, 6.35. The range and median of the ratio in the encephalopathy group were: minimum, 3.62, maximum, 9.04, median, 5.26. One patient in the encephalopathy group was excluded from these results because abnormally high signal intensity was seen diffusely in the right caudate nucleus. Of note, the ratio of this patient was 15.9. The ratio was higher than 3.0 in all cases of both groups. The difference between the groups (the patient with abnormally high signal intensity in the right caudate nucleus was excluded) was not significant (Mann-Whitney test, P = 0.14).

### Results of F_H_ Mapping

**Figure 4:**
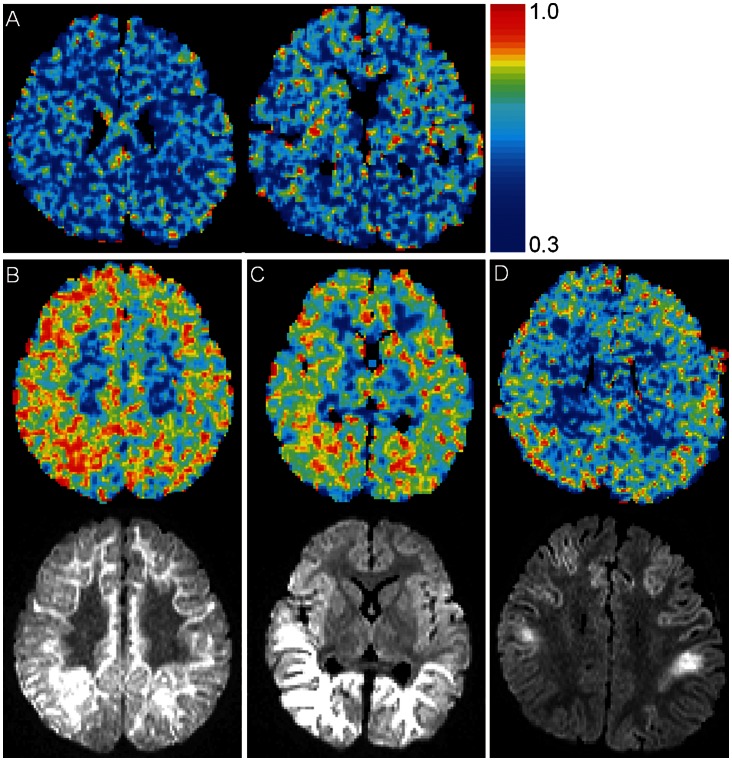
Samples of F_H_ map. A: F_H_ map (F_H_: fraction of high b-pair) calculated from the images acquired from a 2-year-old boy in the control group (the same boy as in Fig. 2 C,D). B, C, and D: F_H_ maps (upper) and corresponding diffusion-weighted images (DWI) at b = 1500 (lower) of 1-year-old girl with unspecific encephalopathy (B), 1-year-old boy with acute encephalopathy with biphasic seizures and late reduced diffusion (AESD) (C), and another 1-year-old boy with AESD (D). F_H_ seems elevated in the cortical and subcortical areas of the encephalopathy group (B, C, and D) compared to the control group (A). The prominence of elevation in F_H_ seems related to the prominence of the abnormally high signal intensity in DWI, but the elevations of F_H_ are seen even in areas where there are no particular findings in DWI (e.g., posterior area of (D)).

Some samples of F_H_ maps are shown in Fig. 4. The masking procedure worked well to abstract the approximate area of brain parenchyma. The map image was relatively noisy; however, the cortex and subcortical area of the patients in the encephalopathy group showed diffusely elevated F_H_ (color closer to red) compared to those in the control group (Fig. 4). The prominence of the elevation in F_H_ seemed to be related to the prominence of the abnormal signal intensity of DWI, but elevations of F_H_ were seen even in areas where there were no particular findings in DWI.

### Results of ROI-based Study

The numbers of ROIs corresponding to each category of each patient of the encephalopathy group are shown in Table 2 (visual assessment). Of note, the categorization of 143 ROIs of 150 ROIs (10 ROIs each in 15 patients) was matched between the interpreters (95.3%), and the other 7 unmatched ROIs were decided by their consensus. The range and median of the number of ROIs categorized as “severe”, “mild”, and “indistinct” in each patient were 0–10 (4), 0–5 (1), and 0–10 (5), respectively (minimum–maximum, median in parentheses).

**Table 2:** Categorization of ROIs of each patient in encephalopathy group.

				Number of ROIs		
Patient	age	sex	diagnosis	Severe	Mild	Indistinct
1	1	M	AESD	0	4	6
2	1	M	AESD	4	4	2
3	1	M	UC	5	3	2
4	1	F	ANE	10	0	0
5	10	F	UC	1	0	9
6	3	F	UC	3	5	2
7	2	M	UC	10	0	0
8	3	M	UC	1	2	7
9	1	F	AESD	1	1	8
10	1	M	UC	0	1	9
11	7	M	UC	0	0	10
12	1	F	UC	4	1	5
13	1	F	AESD	4	2	4
14	1	M	AESD	5	0	5
15	1	F	UC	10	0	0

ROIs: regions of interest, AESD: acute encephalopathy with biphasic seizures and late reduced diffusion, ANE: acute necrotizing encephalopathy, UC: uncategorizable.

The correlation coefficient between F_H_ and patient age in the control group was 0.29, which was not significant (P = 0.28). The correlation coefficient between ADC_0–1500_ and patient age was −0.44, which indicated a negative tendency but without significance (P = 0.08). ADC_500–1500_ was negatively correlated with age (r = −0.57) to a significant extent (P = 0.02). ADC_0–500_ and ADC_1500–2500_ were not significantly correlated with patient age (P = 0.66 and 0.11, respectively).

Mean F_H_ in each category of the encephalopathy group (severe, mild, and indistinct) was higher than that of the control group, with all differences being statistically significant (P<0.05, corrected). On the other hand, mean ADC_0–1500_ was also lower in all three categories of the encephalopathy group compared to the control group, but significant differences were only found in the “severe” and “mild” categories. There was no significant difference between the “indistinct” category and the control group (P = 0.223, corrected) (Table 3).

**Table 3:** Statistical analysis of F_H_ and ADC.

	Control	Encephalopathy		P-value b
F_H_	0.735 (0.028)	Severe	0.810 (0.052)	<0.001
		Mild	0.773(0.033)	0.038
		Indistinct	0.765 (0.028)	0.015
ADC_0–1500_ a	0.879 (0.029)	Severe	0.645 (0.171)	<0.001
		Mild	0.838 (0.056)	0.027
		Indistinct	0.866 (0.050)	0.223
ADC_0–500_ a	1.097 (0.055)	Severe	0.804 (0.217)	<0.001
		Mild	1.021 (0.095)	0.111
		Indistinct	1.048 (0.069)	0.063
ADC_500–1500_ a	0.770 (0.029)	Severe	0.565 (0.150)	<0.001
		Mild	0.746 (0.047)	0.054
		Indistinct	0.774 (0.045)	1.477
ADC_1500–2500_ a	0.564 (0.040)	Severe	0.424 (0.120)	<0.001
		Mild	0.574 (0.045)	2.697
		Indistinct	0.595 (0.037)	0.533

Data denote the averages of the values, and standard deviations are in parentheses.

a ×10^−3^ mm^2^/sec;

b Analysis of covariance between the control and encephalopathy groups (F_H_, ADCs, and patient age as a variable); P-values were tripled according to the correction of Bonferroni. F_H_: fraction of high b-pair, ADC: apparent diffusion coefficient; the pair of subscripted numbers indicate the pair of b-values used to calculate the value.

**Figure 5:**
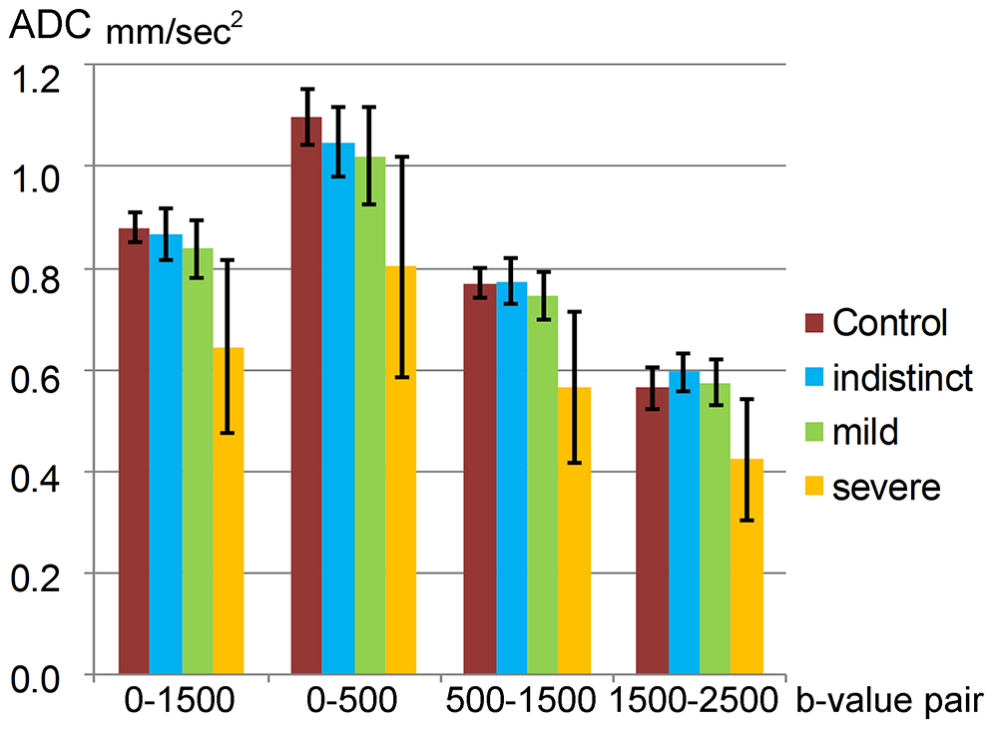
ADCs of each category in each b-value pair. The bar graph shows the mean apparent diffusion coefficient (ADC) in each b-value pair in each category of the encephalopathy group. The error bar indicates standard deviations. The average ADC of the “indistinct” group was lower compared to the control group in the low b-pair (b = 0, 500), but it reversed toward the higher average in the high b-pair (b = 1500, 2500).

Mean ADCs in the “severe” category were always lower than that of the control group with a significant difference regardless of the applied pair of b-values (b = 0 and 500, 500 and 1500, and 1500 and 2500); however, comparing the “indistinct” category and the control group, the mean ADC of the former tended to be lower (P = 0.063, corrected) in the low b-pair (b = 0, 500), but became even higher in the high b-pair (b = 1500, 2500), though the difference was not significant (Table 3, Fig. 5). Mean ADC of the “mild” category was between that of the “severe” and “indistinct” categories in each of the corresponding b-pairs. It was slightly higher than that of the control group in the high b-pair (b = 1500, 2500), but the difference was not significant (Table 3, Fig. 5).

## Discussion

The SNR of the images obtained by the 4bDWI imaging sequence seems enough for the assessments even at b = 2500. In addition, difference between the control and encephalopathy groups was not significant. We evaluated the ratio of the mean signal intensity of b = 2500 and the standard deviation of the signal intensity of b = 0 instead of comparing the average signal intensity of b = 2500 directly with the background noise as usual. This was because it was difficult to evaluate the background noise accurately, since GRAPPA (a parallel imaging technique) was applied in the 4bDWI sequence. However, the ratio we assessed should be stricter than the “usual” SNR because the inhomogeneity of the caudate nucleus at b = 0 was also recognized as a noise in addition to the real noise in this method. Furthermore, the ROI was set near the center of the brain where the SNR is usually not good because of the distance from the coil. Thus the regions assessed in this study (cortex and subcortical white matter) might have had higher SNRs. MR images in this study were acquired with either of two different coil sets depending on the patient’s head size and age, but this we may not need consideration in this study because both obtained images with sufficient SNRs, and the differences between the groups were not significant.

The signal intensity of DWI was obtained only in four different b-values in this study, as the data were collected from the clinical database retrospectively. For this reason, it was inappropriate to fit the data directly to the biexponential equation (Introduction, Eq. 2). We calculated D_f_, D_s_, and f_s_ from S_0_, S_500_, S_1500_, and S_2500_ by least-squares method (data not shown), but they had relatively large divergence, and agreements with those previously reported were poor [18]–[21], [26]. In addition, the data failed to show a significant difference between the control and encephalopathy groups not only in the “indistinct” category but even in the “mild” category.

F_H_ in this study was designed as a value related to the two-compartment model of water diffusion in vivo that could be obtained from this small number of b-values. It was calculated by the theoretical signal intensity of b = 0 obtained from the signal intensity of the higher b-value pair (S_1500_, S_2500_) divided by the theoretical signal intensity of b = 0 calculated by the lower b-value pair (S_500_, S_1500_) (Fig. 1). The former value indicates the signal intensity at b = 0, in a theoretical case when there was only a “slower” diffusion component that could not have been totally attenuated by the b-value of 1500. On the other hand, the latter indicates the signal intensity at b = 0 when “faster” and “slower” diffusion components are both included. In addition, the effect of perfusion may not be included in this “slower” component because its signal might be attenuated at b = 500. Thus, F_H_ indicates the fraction of the “slower” diffusion component against the total diffusion except the very fast diffusion component including perfusion. This value seemed to have a positive correlation with f_s_ in the estimated range of D_s_, D_f_, and f_s_ in vivo (see Text S1). In addition, it also revealed a positive correlation with D_s_, and a negative correlation with D_f_ at the same settings, a point that will be discussed later.

If there were more data points from a larger variation of the b-value, then D_f_, D_s_, and f_s_ could be more directly and accurately calculated by use of the least-squares method. However, more b-values would have required more scan time and more consistent sedation for juveniles. This was not suitable for clinical examination, especially for those with acute severe condition such as the acute encephalopathy discussed in this study. Thus, it stands to reason that, a simple method for assessing the two-compartment model should be the focus of discussion for applying this model in clinics; F_H_ was calculated from data that require only a clinically acceptable scan time, approximately 3 minutes, which in this regard might be a distinct advantage.

The correlation between ADC_500–1500_ and patient age was significant in the control group, but the other values including F_H_ and ADC_0–1500_ (necessarily assessed in this study as a representative ADC used in clinics) showed no significant correlation between ages. However, considering the fact that patient age in the control and encephalopathy groups did not match (P = 0.01, Mann-Whitney test; details not shown), ANCOVA was applied for further statistical comparison to set patient age as an additional variable. Bonferroni correction was applied to avoid type 1 error.

The F_H_ map in this study (Fig. 4) revealed a diffusely increased F_H_ in the cortical and subcortical areas of the encephalopathy group patients. Furthermore, statistical comparison revealed that F_H_ achieved a significant difference between the control group and all three categories of the encephalopathy group (severe, mild, and indistinct). A probable mechanism for the difference may be a change in f_s_. The exact mechanisms underlying the link between the diffusion parameters above (D_s_, D_f_, and f_s_) and tissue microstructure remain largely unknown; however, one of the most accepted assumptions is that D_s_ and D_f_ provide intracellular and extracellular ADCs respectively, and thus f_s_ indicates the fraction of intracellular volume [19]. Non-inflammatory brain edema and decrease in the size of extracellular space owing to cell (especially astrocyte) swelling have been pointed out in acute encephalopathy [29], [30], which may in this respect increase f_s_.

F_H_ demonstrated a significant difference between the control and encephalopathy groups even in the “indistinct” category, while ADC_0–1500_ (the ADC value usually used in clinics) did not show a significant difference in the same comparison. This result may indicate the higher detectability of F_H_ compared to ADC_0–1500_ regarding the less severe pathologic lesions of acute encephalopathy in the cortical and subcortical areas. However, our simulation (Text S1) showed that not only F_H_ but also ADC_0–1500_ reflects the change in f_s_ to some extent (Fig. S1; A, B in Text S1,). One mechanism that possibly contributed to the superiority of F_H_ compared to ADC_0–1500_ may be the increase of D_s_ in the slight change in acute encephalopathy (corresponding to the “indistinct” category). The increase in f_s_ and D_s_ synergistically increases F_H_ (both have a positive correlation with F_H_, as described above) but works antagonistically in ADC_0–1500_, and thus F_H_ has an advantage over ADC_0–1500_ in this setting (Fig. S1 in Text S1). The fact that ADCs of the “indistinct” category gradually become larger than the control group as the b-values enlarge (Table 3, Fig. 5) may at least slightly support this hypothesis. This is because D_s_ may be reflected more in the ADC of high b-pairs; the elevation of f_s_ may have a smaller effect in ADC of higher b-pairs, as the signal from the fast diffusion component is generally relatively attenuated by the motion proving gradient in high b-values, so that the importance of f_s_ might be limited, whereas the change in D_s_ might be greatly reflected in ADC of higher b-pairs. One possible mechanism for increasing D_s_ is the increased expression regulation of aquaporin-4 (AQP4). Recently, the relation of AQP4 regulation and brain edema was discussed in various pathologic states in mouse and human [31]–[34]. Up- or down-regulation of AQP4 seems quite complicated, so its mechanism is still not clear, although some of the aforementioned reports revealed an up-regulation of AQP in acute brain lesions [32], [33]. Obata et al. discussed decreased D_s_ in the AQP knockout cell [35], which may contrarily indicate increased D_s_ in the up-regulated expression of AQP4. The relationship between pediatric acute encephalopathy and AQP4 is not yet well understood, so further studies will be required to confirm this hypothesis.

Another possible mechanism for the increase of D_s_ is the effect of a fever, which was more frequently seen in the encephalopathy group. It is well known that higher temperature induces more active molecular movement, which increases water diffusion. Recently, thermometry of CSF in the use of diffusion-weighted imaging has been well discussed [36]–[38]. Following the method discussed in the previous report [36], the diffusion coefficient of CSF in the lateral ventricle may increase by approximately 7% when body temperature increases from 36 to 38°C (2.9676 to 3.1102 mm/sec^2^). Changes in D_s_ and D_f_ in hyperthermia have not been discussed, but it is conceivable that they may increase to some extent. Under such condition, ADC_1500–2500_ may increase, as it reflects D_s_.

In this respect, ADC_0–1500_, ADC_0–500_, and ADC_500–1500_ should also have increased by the effect of increase in D_s_, but the simultaneous increase in f_s_ (which is an increased fraction of a much slower diffusion (e.g. D_s_) than D_f_ even if elevated as discussed), may have excessively worked antagonistically and thereby concealed this effect; ADC_1500–2500_ might be less affected by the increased f_s_, as it is related to D_s_ more directly than the other ADCs for the high b-values, so the effect from the change in the fraction of D_s_ (e.g. f_s_) may be relatively limited.

Of note, it may be important that a febrile state may work anyway, antagonistically, for detecting diffusion restriction in the use of ADC, because the method is very commonly used in clinics regardless of this issue.

There was not enough data in this study to allow the evaluation of the detailed changes in D_s_, and further study will be necessary. In addition, we may note that the ADC_0–500_ discussed above might be affected by perfusion. Another aspect of the result is that there may be some pathologic change in the encephalopathy group even in areas of unclear imaging findings. This suggests that acute encephalopathy is a pathologic condition of the whole brain, which well illustrates the discrepancy often experienced between the imaging findings and the clinical outcome – imaging findings do not always correspond exactly to the responsible area of impaired function. Recently, a mutation of the SCN1A gene (codes neuronal sodium channel alpha 1 subunit) was found to be the predisposing factor for the onset of various types of acute encephalopathy [14], [17]. In addition, another report discussed genetic seizure susceptibility [16]. Pathologic mediation by cytokines has also been discussed in some cases of acute encephalopathy [2], [39], [40]. These non-focal factors may have caused diffuse alteration in water diffusion of the whole brain.

Our study has some limitations. It was a retrospective study with a relatively small sample size. In addition, selection was biased because 4bDWI was obtained only when the need was determined by an experienced radiologist, and excluding bias was difficult because all study imaging was performed as part of clinical care. Another limitation is that we discussed the encephalopathy cases altogether, regardless of their various clinical patterns that might have included different pathologies. However, as the pathologic mechanisms of acute encephalopathies are not well known, and approximately half of the cases in this study were non-specific, a more detailed grouping was difficult. Further studies with typical specific cases are necessary. In addition, further study and understanding of the pathogenesis of acute encephalopathies may be important. The last limitation is that the correlation between F_H_ and the two-compartment model, as well as the histological background to change in F_H_, was not experimentally confirmed in this study. Further study may also be required concerning this point.

### Conclusion

The results of the present study suggest that F_H_ is superior to ADC in detecting slight changes in cortical and subcortical white matter lesions in pediatric acute encephalopathy.

## Supporting Information

Text S1 Appendix with fig. S1; Theoretical correlation between F_H_, ADC and the two-compartment model.
(PDF)
