# Supplementary material for: Analysis of Multiple B-Value Diffusion-Weighted Imaging in Pediatric Acute Encephalopathy
Source: PLoS One. 2013 Jun 3;8(6):e63869. doi: 10.1371/journal.pone.0063869 (PMC3670889; doi:10.1371/journal.pone.0063869)
Supplement: Text S1 — Appendix with fig. S1; Theoretical correlation between FH, ADC and the two-compartment model. (PDF) [file pone.0063869.s001.pdf]

Appendix

To assess how F_H and $ADC_{0\text{-}1500}$ change when D_f, D_s, and f_s in the two-compartment model change, simulation using a theoretical model of human brain was performed. The model signal intensities of b=0, 500, 1500, 2500 were calculated from Eq.2 (see Introduction), in which D_f, D_s and f_s were altered between 1.10 and 1.50 $x10^{-3}mm/sec^2$, 0.10 and 0.50 $x10^{-3}$ mm/sec^2, and 0.2 and 0.6 respectively. S_0 was fixed at 1. These values were set according to a previous article on water diffusion in normal brains (white matter, gray matter, and basal ganglia), stroke, and brain tumors [1-5]. Then, F_H and $ADC_{0\text{-}1500}$ were calculated from this simulated signal intensity for each altered D_s and f_s, respectively.

From these data, changes in F_H and $ADC_{0\text{-}1500}$ were compared in the case in which f_s and D_s changed between the aforementioned ranges (D_f was fixed to the median of the range) (Fig. 6; A, B). The change in F_H when f_s was fixed to the median of the range and D_s and D_f altered between the ranges was also assessed (Fig. 6; C).

In this setting, F_H had a positive correlation with both f_s and D_s. $ADC_{0\text{-}1500}$ had a negative correlation with f_s and a positive correlation with D_s (Fig. A; A, B). Thus, increase in both f_s and D_s works synergistically to increase F_H and works antagonistically to $ADC_{0\text{-}1500}$ in this setting.

References

1. Maier SE, Mulkern RV (2008) Biexponential analysis of diffusion-related signal decay in normal human cortical and deep gray matter. Magn Reson Imaging 26: 897-904.

2. Maier SE, Bogner P, Bajzik G, Mamata H, Mamata Y, et al. (2001) Normal brain and brain tumor: multicomponent apparent diffusion coefficient line scan imaging. Radiology 219: 842-849.

3. Clark CA, Le Bihan D (2000) Water diffusion compartmentation and anisotropy at high b values in the human brain. Magn Reson Med 44: 852-859.

4. Brugieres P, Thomas P, Maraval A, Hosseini H, Combes C, et al. (2004) Water diffusion compartmentation at high b values in ischemic human brain. AJNR Am J Neuroradiol 25: 692-698.

5. Mulkern RV, Gudbjartsson H, Westin CF, Zengingonul HP, Gartner W, et al. (1999) Multi-component apparent diffusion coefficients in human brain. NMR Biomed 12: 51-62.

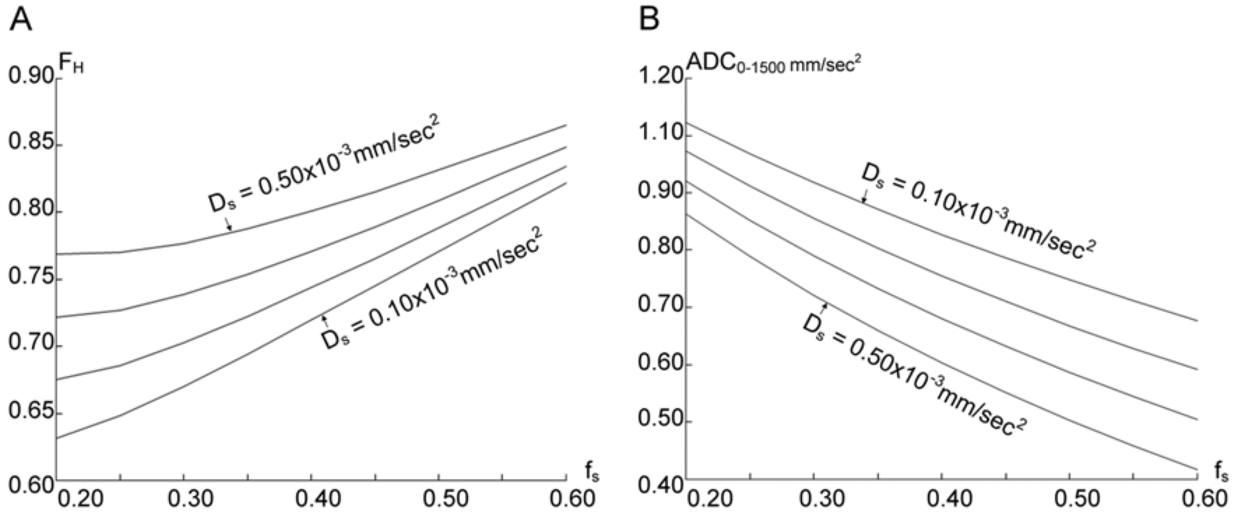

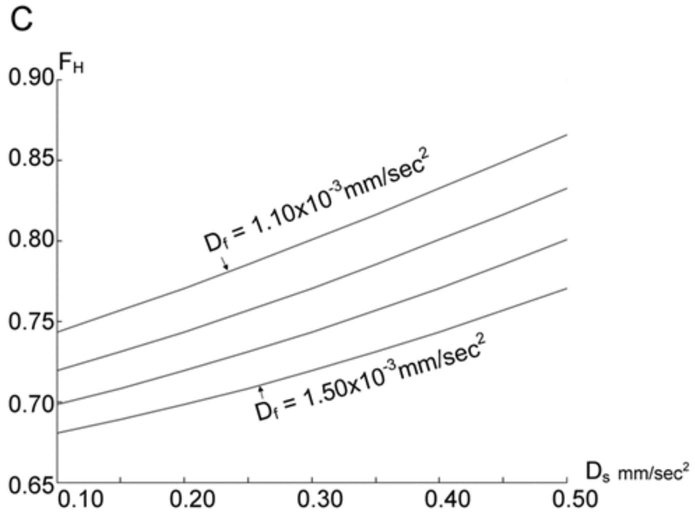

Figure 6. Theoretical correlation between F_H, ADC and the two-compartment model.

A, B) The graphs indicate the positive correlation of the fraction of high b-pair (F_H) and the negative correlation of apparent diffusion coefficient (ADC) calculated from the signal intensities of b=0 and 1500 ($ADC_{0\text{-}1500}$) with the fraction of the slow diffusion component (f_s). Different lines in each graph represent different ADCs of the slower diffusion component (D_s), which had a positive correlation with both F_H and $ADC_{0\text{-}1500}$. C) The graph indicates the increase in F_H as D_s increases, and the decrease in F_H as D_f increases (different lines indicate different diffusion coefficients of the fast diffusion component (D_f)).
